# AXL receptor tyrosine kinase modulates gonadotropin-releasing hormone receptor signaling

**DOI:** 10.1186/s12964-023-01313-y

**Published:** 2023-10-12

**Authors:** Pardis Mohammadzadeh, Mina Roueinfar, Gregory C. Amberg

**Affiliations:** Department of Biomedical Sciences, Colorado State University, 1617 Campus Delivery, Fort Collins, CO 80523 USA

**Keywords:** TAM receptor tyrosine kinase, Gas6, Matrix metalloproteinase 9

## Abstract

**Background:**

Gonadotropin-releasing hormone (GnRH) receptors are essential for reproduction and are expressed in numerous urogenital, reproductive, and non-reproductive cancers. In addition to canonical G protein-coupled receptor signaling, GnRH receptors functionally interact with several receptor tyrosine kinases. AXL is a receptor tyrosine kinase expressed in numerous tissues as well as multiple tumors. Here we tested the hypothesis that AXL, along with its endogenous ligand Gas6, impacts GnRH receptor signaling.

**Methods:**

We used clonal murine pituitary αT3-1 and LβT2 gonadotrope cell lines to examine the effect of AXL activation on GnRH receptor-dependent signaling outcomes. ELISA and immunofluorescence were used to observe AXL and GnRH receptor expression in αT3-1 and LβT2 cells, as well as in murine and human pituitary sections. We also used ELISA to measure changes in ERK phosphorylation, pro-MMP9 production, and release of LHβ. Digital droplet PCR was used to measure the abundance of Egr-1 transcripts. A transwell migration assay was used to measure αT3-1 and LβT2 migration responses to GnRH and AXL.

**Results:**

We observed AXL, along with the GnRH receptor, expression in αT3-1 and LβT2 gonadotrope cell lines, as well as in murine and human pituitary sections. Consistent with a potentiating role of AXL, Gas6 enhanced GnRH-dependent ERK phosphorylation in αT3-1 and LβT2 cells. Further, and consistent with enhanced post-transcriptional GnRH receptor responses, we found that Gas6 increased the abundance of Egr-1 transcripts. Suggesting functional significance, in LβT2 cells, Gas6/AXL signaling stimulated LHβ production and enhanced GnRH receptor-dependent generation of pro-MMP9 protein and promoted cell migration.

**Conclusions:**

Altogether, these data describe a novel role for AXL as a modulator of GnRH receptor signaling.

Video Abstract

**Supplementary Information:**

The online version contains supplementary material available at 10.1186/s12964-023-01313-y.

## Background

Reproductive processes are largely governed by hormones produced by the hypothalamic-pituitary–gonadal (HPG) axis. Secretory hypothalamic neurons release gonadotropin-releasing hormone (GnRH) into the hypophyseal portal circulation which delivers the neuropeptide to the anterior pituitary. A small population of endocrine cells called gonadotropes respond to the released GnRH via binding to cognate receptors expressed on their cell surface. GnRH receptor activation initiates several signaling cascades which culminate in the synthesis and secretion of the gonadotropins luteinizing hormone (LH) and follicle stimulating hormone (FSH) into the general circulation [1]. Circulating gonadotropins subsequently bind and activate receptors in the gonads to promote gametogenesis and synthesis of sex steroid hormones.

During development GnRH neurons arise in the olfactory placodes and migrate to the forebrain [2]. Mislocalization of GnRH neurons due to failed or impaired migration, or loss by some other mechanism, is associated with delayed or an absence of puberty [3, 4]. The receptor tyrosine kinase AXL, a member of the TAM family of receptor tyrosine kinases (Tyro3, AXL, and Mer), is involved with GnRH neuronal migration and survival [5, 6]. AXL receptors are activated by the high-affinity ligand Gas6 (growth arrest-specific protein 6) and by ligand-independent mechanisms [7, 8]. Gas6/AXL signaling is implicated in the development of a variety of tumors by promoting cell survival, proliferation, migration, and invasion [9]. As such, AXL receptors are currently a promising target for several novel cancer chemotherapeutics [10, 11].

Following activation, intrinsic AXL tyrosine kinase activity phosphorylates C-terminal tyrosine residues which leads to recruitment and activation of multiple intracellular signaling proteins [12–16]. Examples include phosphatidylinositol 3-kinase (PI3K), Src tyrosine kinase, phospholipase C-γ, protein kinase C (PKC), Rho GTPases, matrix metalloprotease 9 (MMP9), and mitogen-activated protein kinases (MAPK). AXL signaling is further diversified by functional coupling with other receptor tyrosine kinases such as epidermal growth factor receptor (EGFR) and platelet derived growth factor receptor (PDGFR) [17–19]. AXL receptors couple to the extracellular signal-regulated kinase (ERK) branch of MAPK signaling pathways [10, 20]. MAP kinases transduce extracellular signals from plasmalemmal receptors into changes in cellular function such as differentiation, survival, and migration. Indeed, Gas6/AXL receptor promotion of the survival and migration of GnRH neurons during development involves ERK signaling-dependent processes [21].

In gonadotropes, activation of GnRH receptors leads to canonical Gα_q_ protein signaling where phospholipase C (PLC) cleaves phosphatidylinositol-4–5-bisphosphate (PIP_2_) to generate the classic second messengers inositol-1,4,5-trisphosphate (IP_3_) and diacylglycerol (DAG) [22]. IP_3_ induces calcium release from the endoplasmic reticulum (ER) while DAG stimulates protein kinase C (PKC) which ultimately increases calcium influx through voltage-dependent channels [23]. Calcium release from the ER then promotes c-Jun N-terminal kinase (JNK) signaling to increase FSH expression while calcium influx through voltage-dependent channels activates ERK signaling to increase LH expression [23–25]. In addition, GnRH receptor signaling to ERK in gonadotropes has been shown to incorporate MMP9 activation [26]. As noted above, AXL receptor activation also promotes ERK- and MMP9-dependent signaling processes [16]. Despite sharing several common downstream signaling mechanisms, the impact of crosstalk between AXL and GnRH receptors is unclear.

In this study we tested the hypothesis that Gas6/AXL and GnRH receptors coordinate to produce GnRH-dependent signaling outcomes. After establishing Gas6 and AXL receptor expression in human and murine pituitary slices, we used clonal murine gonadotropes to investigate the functional consequences of AXL receptor activation on GnRH receptor function. We found that Gas6-dependent AXL activation enhances downstream GnRH receptor processes including ERK activation, transcriptional responses, luteinizing hormone (LH β) generation, pro-MMP9 levels, and cell migration. Taken together, our data supports the concept of AXL receptors as novel modulators of GnRH receptor-dependent regulation of gonadotrope function.

## Methods

### Reagents

Cell culture reagents were from Thermo Fisher Scientific (Waltham, MA) and chemicals were from Sigma (St. Louis, MO) unless noted otherwise. Test reagents used include: R428 (APExBio; Houston, TX), cetrorelix acetate (Tocris Bioscience; Minneapolis, MN), Gas6 (Novus Biologicals; Centennial, CO), GnRH (Sigma), and U0126-EtOH (APExBio; Houston, TX). All drugs used were screened for potential cell toxicity at 3 and 24 h with a tetrazolium-based colormetric XTT Assay Kit (Abcam; Boston, MA). All drugs used were found to be non-toxic at the concentrations used.

### Cell culture

Clonal LβT2 and αT3-1 (kindly provided by Dr. Pamela Mellon, University of California, San Diego) gonadotrope cells were cultured in Dulbecco’s Modified Eagle Medium (DMEM) supplemented with 10% (v/v) Fetal Bovine Serum and 1% (v/v) Antibiotic–Antimycotic and maintained at 37 °C in 5% CO_2_ humidified air. All experiments were performed on low passage number (6 to 8) αT3-1 and LβT2 cells.

### Immunofluorescence

LβT2 or αT3-1cells (2 × 10^5^ cells/well) were plated onto 35 mm glass bottom dishes (Matsunami Glass; Bellingham, WA) coated with Geltrex (Thermo Fisher Scientific). Cells were cultured overnight in 2 mL media (as above) and, on the day prior to fixation, media were replaced with 2 mL of media containing 10% charcoal stripped FBS. The next day, cells were treated with agonists/antagonists for various times plus 2 µl CellMask Deep Red Actin Tracking Stain (Invitrogen; Waltham, MA) for 30 min.

Following completion of experiments, cells were washed with 1X D-PBS with calcium and magnesium three times and fixed in freshly prepared 4% paraformaldehyde in PBS. After fixation, cells were washed with 1X PBS three times, and blocked in eBioscience IHC/ICC Blocking Buffer—High Protein (Thermo Fisher Scientific) for 30 min. Samples were then incubated overnight at 4 °C with GnRHR Monoclonal primary antibody (GNRHR/768, ab220196, Abcam) at 1 µg/mL dilution and AXL primary Antibody (PA5-106,118, Thermo Fisher Scientific) at 1:200 dilution in blocking buffer. The next day, cells were washed three times with 1X eBioscience TBS Wash Buffer for IHC/ICC (Thermo Fisher Scientific) and Alexa Fluor 546 (A-11035, Thermo Fisher Scientific) along with Alexa Fluor 488 (A-11029, Thermo Fisher Scientific) diluted 1:500 in blocking buffer, were added to the cells for 1 h at room temperature. Finally, nuclei were stained, and cells were mounted with ProLong Glass Antifade Mountant containing NucBlue Stain (Thermo Fisher Scientific) following the manufacture’s protocol.

C57 murine (male and female) and Human (unidentified) pituitary 5 µm paraffin sections were purchased from Zyagen (San Diego, CA) and immunostained for AXL, GnRH receptor (GnRHR) and Gas6 proteins [27]. Briefly, tissues were de-waxed using five-minute washes in CitriSolv (Decon Labs, 1601), then rehydrated through a gradual alcohol series for 5-min each (100%, 90%, 70%, 50% and 30% ethanol) and finally were washed in deionized water for two minutes. Afterwards, the tissue sections were microwaved in citric acid-based Antigen Unmasking Solution (pH 6.0; Vector Laboratories, H-3300–250) for 20 min. Slides were washed for five minutes in 1 × PBS and incubated for twenty minutes followed by incubation with the quenching solution in a humidity chamber for 5 min using Vector Trueview Autofluorescence Quenching Kit (Vector Laboratories).

To prevent nonspecific binding, tissue sections were incubated for thirty minutes with eBioscience IHC/ICC Blocking Buffer—High Protein (Thermo Fisher Scientific). After blocking, tissue sections were incubated with GnRHR monoclonal antibody (GNRH03, Thermo Fisher Scientific) at 1:100 dilution, AXL antibody (PA5-77,875, Thermo Fisher Scientific) at 1:200 dilution, AXL antibody (PA5-106,118, Thermo Fisher Scientific) at 1:200 dilution, GnRHR monoclonal antibody (GNRHR/768, ab220196, Abcam) at 1 µg/mL dilution, Gas6 rabbit antibody (A8545, ABclonal) at 1:100 dilution and Gas6 goat antibody (AF885SP, R&D Systems) at 5 µg/mL dilution in blocking buffer in a humidified chamber at 4 °C overnight. The following day, sections were washed three times (5 min each) in 1X eBioscience TBS Wash Buffer for IHC/ICC (Thermo Fisher Scientific) and incubated for with appropriate secondary antibodies (Alexa Fluor 546 (A-11035, Thermo Fisher Scientific), Alexa Fluor 488 (A-11029, Thermo Fisher Scientific and NC0679377, Abcam)) all at 1:500 dilution in blocking buffer for 1 h at room temperature. The slides were then washed in 1 × PBS three times (5 min each) and the tissues dehydrated using a gradual alcohol series (50%, 70%, 90% and 100%). Finally, cells were mounted, and the nuclei were stained with ProLong Glass Antifade Mountant with NucBlue Stain (Thermo Fisher Scientific) following the manufacture’s protocol. Slides were later imaged at 40X and 63X using an LSM 800 Airyscan (Zeiss) confocal microscope and processed via Zen Blue software (Zeiss).

### AXL protein quantification

LβT2 and αT3-1 cells (2 × 10^5^ cells/well) were seeded in 6-well plates and incubated for 48 h after which GnRH (10 nM) was added for 24 h. Protein was then extracted using RIPA Lysis and Extraction Buffer (Thermo Fisher Scientific) containing Halt Protease and Phosphatase Inhibitor Cocktail (Thermo Scientific). Total protein was measured with Rapid Gold BCA Protein Assay Kit (Thermo Fisher Scientific) and AXL protein was quantified with a murine AXL ELISA kit following the manufacturer’s instructions (Thermo Fisher Scientific). Each independent experiment was performed in duplicate.

### AXL and GnRH receptor distribution in human pituitary

Images of human pituitary stained for AXL (red), GnRHR (green) and nucleus (blue) were used to identify the expression and distribution pattern of AXL and GnRHR throughout the pituitary. Using Zen blue software, a total of 577 cells were masked in one 2 × 2 tiled and two regular images. Images were then processed and fluorescent intensities for TaRFP, AF488 and DAPI channels (corresponding to AXL, GnRHR and nuclei respectively) were extracted. The fluorescent intensities were then divided by the area for each cell (µm^2^) and plotted as histograms.

### ERK phosphorylation measurements

Semiquantitative sandwich ELISA kit (Abcam, ab176660) was used to measure ERK phosphorylation (pERK) and total ERK (pERK/ERK) in LβT2 and αT3-1 cells in response to Gas6 (100 nM) at different time points and GnRH (10 nM, 5 min) was used as a positive control. Briefly, cells were seeded at 8 × 10^4^ cells/well in 6-well plates and incubated for 2 days until 60–70% confluent. Cells then were washed with 1X PBS two times and media was changed to 2 mL/well charcoal stripped DMEM and incubated at 37 °C in 5% CO_2_ humidified air over night (12 h). Cells were then treated with Gas6 and/or GnRH for 5 min to 2 h; cell extracts were then obtained with RIPA Lysis and Extraction Buffer (Thermo Scientific) containing Halt Protease and Phosphatase Inhibitor Cocktail (Thermo Scientific) was collected in separate tubes. BCA assay was performed to determine protein concentrations prior to ELISA analysis according to the manufacture’s protocol.

### Reverse transcription digital droplet PCR

Reverse transcription digital droplet PCR (RT-ddPCR) was used to measure absolute transcript levels of the immediate early gene Egr1. LβT2 and αT3-1 cells were seeded in 6 well plates (2 × 10^5^ cells/well) in DMEM supplemented with 10% FBS and 1% antibiotic–antimycotic. Media were changed after 24 h to DMEM supplemented with 10% charcoal stripped FBS and serum starved overnight. Cells were treated with Gas6, GnRH or Gas6 + GnRH for 0, 5, 10, 20, 40 and 60 min, then collected and preserved in 1X DNA/RNA Shield (Zymo Research; Irvine, CA). Collected RNA was purified with the Quick-RNA Miniprep Plus Kit (Zymo Research) and cDNA was synthesized from 40 ng RNA of each sample using iScript cDNA Synthesis Kit (Bio-Rad; Hercules, CA).

EvaGreen (Bio-Rad) RT-ddPCR was performed on a QX200 system (DG8 cartridge, QX200 droplet generator, PX1 PCR plate sealer, C1000 Touch thermal cycler and QX200 droplet reader (Bio-Rad). Briefly, EvaGreen ddPCR supermix was prepared using 10 ng cDNA, 10 µL EvaGreen, and 5 µM of primers for murine Egr1 (Fwd 5’-GAGCGAACAACCCTATGAGC-3’ and Rev 5’-AGCGGCCAGTATAGGTGATG-3’), and glyceraldehyde 3-phosphate dehydrogenase (GAPDH; Fwd 5’-GGGAAGCCCATCACCATCTT-3’ and Rev 5’-GCCTTCTCCATGGTGGTGAA-3’), plus nuclease-free H_2_O to reach a total of 20 µL, which was then used to generate droplets.

Droplets were transferred into a ddPCR Semi-Skirted 96-Well Plate (Bio-Rad), sealed with Pierceable Foil Heat Seal, and amplified with the following thermal protocol: polymerase activation (initial denaturation) at 95 °C for 5 min, 40 cycles of amplification at 95 °C for 30 s (denaturation) and 60 °C for 1 min (annealing/elongation) with a ramp of 2 °C/s for each step. Following amplification, droplets were stabilized at 4 °C for 5 min followed by 95 °C for 5 min and then an infinite hold at 4 °C. For analysis, the PCR plate containing the droplets was placed in the QX200 droplet reader for data acquisition and absolute quantification analysis with QuantaSoft (Bio-Rad) software. Transcript copy numbers were adjusted post hoc to GAPDH transcript levels for each sample.

### LHβ release measurements

LHβ production and release from LβT2 cells were measured with a murine LH Beta SimpleStep ELISA Kit (Abcam). Briefly, 1 × 10^4^ cells/well were seeded in 96-well plates and incubated for 2 days until 60–70% confluent. Cells then were washed with 1X PBS two times and media were changed to 200 μL/well serum-free DMEM and incubated at 37 °C in 5% CO_2_ humidified air for 2 h. Cells were then treated with GnRHR ligand (GnRH, 10 nM), AXL ligand (Gas6, 100 nM), GnRH receptor inhibitor (cetrorelix acetate, 2 nM), AXL inhibitor (R428, 50 nM) and MEK inhibitor (U0126-EtOH, 10 µM) for 2 h and both cell extracts using RIPA Lysis and Extraction Buffer and media were collected in separate tubes containing Halt Protease and Phosphatase Inhibitor Cocktail (Thermo Scientific). ELISA was done according to the manufacturer’s instructions. Total LHβ was calculated as (LHβ quantity in cell extract + LHβ quantity in the media of the same well). Each individual experiment was performed in duplicate.

### Pro-MMP9 release measurements

Pro-MMP9 production and release from LβT2 and αT3-1 cells were measured with a pro-MMP-9 Murine ELISA Kit (Invitrogen). Briefly, 1 × 10^4^ cells/well were seeded in 96-well plates and incubated for 2 days until 60–70% confluent. Cells then were washed with 1X PBS two times and media were changed to 200 µL/well DMEM containing 10% stripped FBS and incubated at 37 °C in 5% CO2 humidified air for 24 h. Cells were then treated with GnRH (10 nM), AXL ligand (Gas6, 100 nM), GnRH receptor inhibitor (cetrorelix acetate, 2 nM), AXL inhibitor (R428, 50 nM), and MEK inhibitor (U0126-EtOH, 10 µM) for 24 h and both cell extracts using RIPA Lysis and Extraction Buffer and media were collected in separate tubes containing Halt Protease and Phosphatase Inhibitor Cocktail (Thermo Scientific). ELISA was performed following the manufacturer’s instructions.

### Cell migration assay

We measured the migratory response of LβT2 and αT3-1 cells to stimuli with a Cell Migration/Chemotaxis Assay Kit (Abcam). LβT2 and αT3-1 cells were seeded in 100 mm plates at 60–70% confluency in DMEM supplemented with 10% FBS and 1% Antibiotic–Antimycotic. Media were changed after 24 h to DMEM supplemented with 10% charcoal stripped FBS and serum starved for 24 h and then were seeded in the upper chamber of the kit and assembled based on the manufacturer’s instructions. GnRH receptor ligand (GnRH, 100 nM), AXL ligand (Gas6, 1000 nM), combination of Gas6 and GnRH, MEK activator (PAF C-16, 100 µM) and MEK inhibitor (U0126-EtOH, 100 µM) were then added to the upper and bottom chambers of the kit. Cells were then incubated for 48 h with the stimulators/inhibitors and migration percentage was measured following the manufacturer’s instructions.

### Statistical analysis

Figures were created and data were analyzed with GraphPad Prism and MathWorks MATLAB. Two-sample comparisons were performed using either a paired or unpaired (as appropriate) two-tailed Student’s t-test; comparisons between more than two groups were performed using one- or two-way (as appropriate) ANOVA with Dunnett’s multiple comparison post-test. All data are presented as mean ± SEM, with *n* ≥ 3 for all comparisons; *n* indicates the number of independent biological replicates unless stated otherwise. *P* < 0.05 was considered significant and asterisks (*) used in the figures are included to indicate significance; ns = not significantly different (*P* ≥ 0.05).

## Results

To test our hypothesis that AXL receptor activation modulates GnRH receptor function we set four requisite experimental criteria: 1) AXL receptors must be expressed in cells with GnRH receptors; 2) activation of AXL receptors should impact GnRH receptor-dependent signaling processes; 3) AXL activation should alter GnRH receptor-dependent changes in gene expression; and 4) AXL receptor activation must impact GnRH receptor-associated biological functions (e.g., gonadotropin production).

### AXL receptor-like expression in the anterior pituitary

**Fig. 1:**
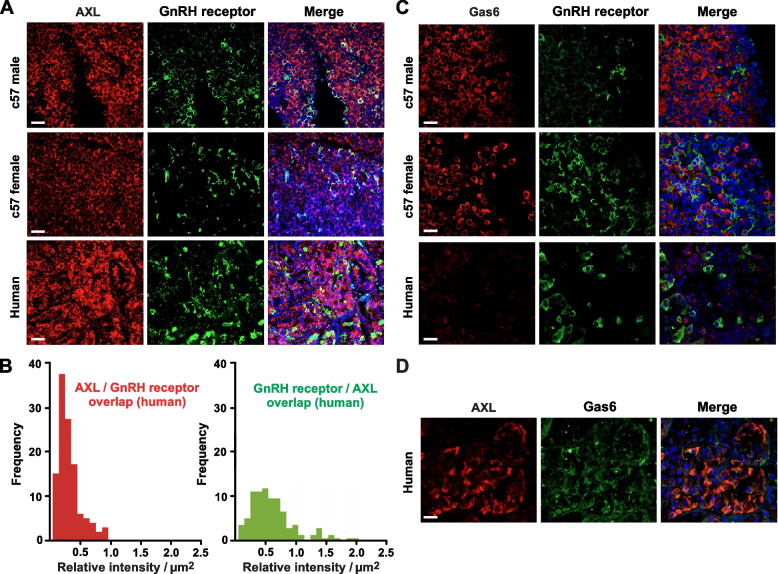
AXL and GnRH receptor-like immunoreactivity in murine and human pituitaries. **A** Representative images showing AXL-like (red) and GnRH receptor-like (green) immunostaining in human, male C57 murine, and C57 female murine pituitary sections. Cell nuclei (blue) blue are shown in the merged images. **B** Frequency of overlapping AXL- and GnRH receptor-like immunoreactivity in a human pituitary; > 100 cells were analyzed from 7 different images for both AXL- and GnRH receptor-like immunoreactivity. **C** Representative images showing Gas6-like (red) and GnRH receptor-like (green) immunostaining in human, male C57 murine, and C57 female murine pituitary sections. Cell nuclei (blue) blue are shown in the merged images. **D** Representative images showing AXL-like (red) and Gas6-like (green) immunostaining in a human pituitary section. Cell nuclei (blue) blue are shown in the merged images. Images are representative of ≥ 3 paraffin sections; scale bars = 20 μm

To begin our investigation of AXL receptor tyrosine kinase regulation of GnRH receptor function, we used immunohistochemistry to assess AXL expression in commercially available murine and human pituitary paraffin sections. Gonadotropes were identified by co-immunostaining for the GnRH receptor. Consistent with AXL expression in gonadotropes, we observed AXL-like and GnRH receptor-like immunoreactivities in murine (C57 male and female) human (sex unknown) pituitary sections (Fig. 1A). Although AXL-like and GnRH receptor-like immunoreactivities were heterogeneous, there was considerable overlap between the signals for the two proteins (Fig. 1B). Indeed, in the human pituitary sections, > 99% of the cells identified as GnRH receptor positive were also positive for AXL-like immunoreactivity. Conversely, ≈ 19% of the cells identified as AXL positive were also positive for GnRH receptor-like reactivity.

To assess expression of the endogenous AXL ligand Gas6, we again performed immunohistochemistry on murine and human pituitary paraffin sections. Along with GnRH receptor-like staining, we observed robust Gas6-like immunoreactivity in murine pituitary sections and weak but distinct Gas6-like immunoreactivity in human pituitary sections (Fig. 1C and D). In contrast to AXL-like immunoreactivity, there was little to no overlap between the Gas6 and GnRH receptor signals in any of the pituitary sections imaged (Fig. 1C, *right*). Therefore, and unlike AXL, Gas6 appears to be expressed in non-gonadotrope cells. Altogether, our immunohistochemical data suggest that in the anterior pituitary, AXL expression is associated with GnRH receptor-positive cells (i.e., gonadotropes) and that the endogenous ligand Gas6 is expressed in surrounding GnRH receptor-negative cells.

### AXL receptor expression in murine gonadotrope cell lines

**Fig. 2:**
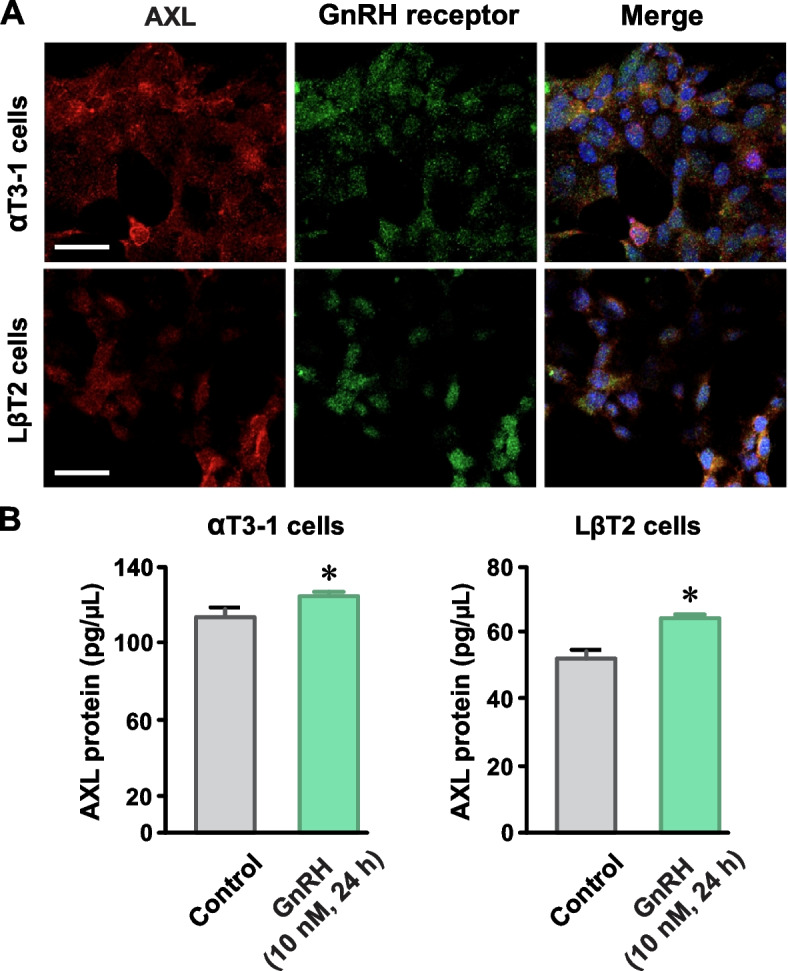
AXL receptor expression in murine gonadotrope cell lines. AXL-like immunoreactivity (**A**) and protein abundance (**B**) in αT3-1 and LβT2 murine gonadotrope cell lines and AXL protein abundance in control cells and cells incubated with GnRH (10 nM for 24 h; *n* = 3 independent cultures). Images representative of ≥ 3 αT3-1 and LβT2 cell cultures; *n* = number of independent experiments; numerical data presented as mean ± SEM; * *P* < 0.05

To investigate the functional interactions between AXL and GnRH receptor function we took advantage of two different murine gonadotrope cell lines. αT3-1 and LβT2 cells, both of which express functional GnRH receptors, are widely used experimental surrogates for native gonadotropes [28]. αT3-1 cells are considered to have a relatively immature gonadotrope phenotype while LβT2 cells represent a more mature form [29, 30]. First, using immunohistochemistry, we confirmed AXL- and GnRH receptor-like immunoreactivity in each cell line. Similar to our results in pituitary sections, we observed overlapping AXL and GnRH receptor signal in αT3-1 and LβT2 cells (Fig. 2A). While the biological significance requires further investigation, using quantitative AXL ELISA we found that incubating αT3-1 and LβT2 cells with GnRH (10 nM) slightly increased AXL protein levels after 24 h (Fig. 2B).

### AXL receptor activation enhances canonical GnRH receptor-dependent signaling processes

**Fig. 3:**
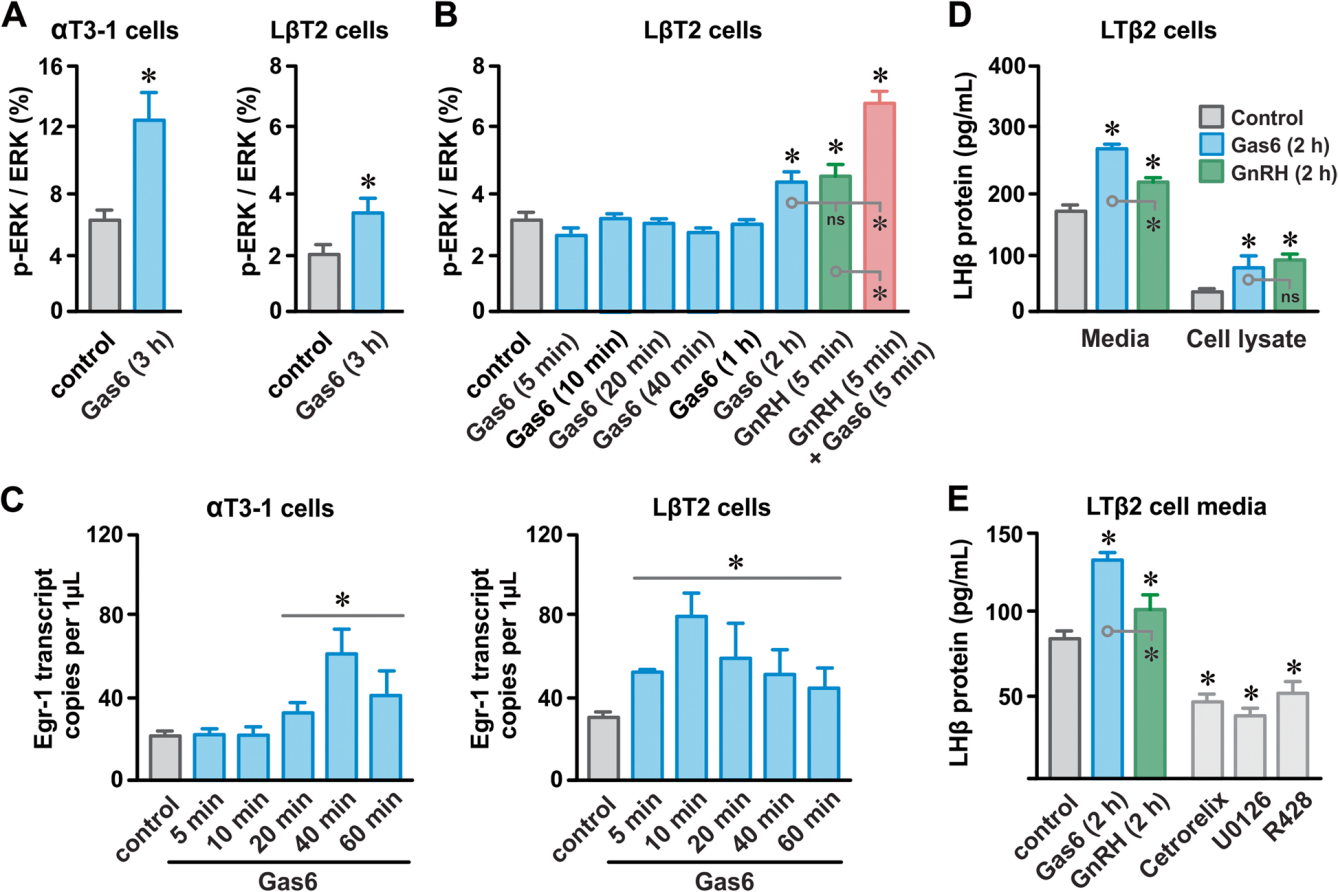
Gas6-dependent AXL receptor activation enhances GnRH receptor signaling processes. **A** Proportion of phosphorylated ERK relative to total ERK (pERK/ERK) in αT3-1 and LβT2 cells under control conditions and after incubation with Gas6 (100 nM for 3 h); *n* = 3 for each condition. **B** Time course of Gas6-dependent changes (100 nM) in pERK/ERK in LβT2 cells (from *left*); effect of incubating LβT2 cells with GnRH (10 nM) and GnRH plus Gas6 (100 nM) for 5 min on pERK/ERK (at *right*); *n* = 3 for each condition. **C** Time course of Gas6-dependent (100 nM) changes on Egr-1 transcript abundance in αT3-1 and LβT2 cells; *n* = 3. **D** LHβ protein abundance in the culture media and lysates of LβT2 cells under control conditions and after 2 h incubations with Gas6 (100 nM) and GnRH (10 nM); *n* = 3. € LHβ protein abundance in LβT2 culture media under control conditions and after 2 h incubations with Gas6 (100 nM), GnRH (10 nM), the GnRH receptor antagonist cetrorelix (2 nM), the AXL receptor antagonist R428 (50 µM), and the MEK inhibitor U0126 (10 µM); *n* = 3 for each condition. *n* = number of independent experiments; numerical data presented as mean ± SEM; * *P* < 0.05

Gonadotrope GnRH receptor activation promotes MAP kinase-dependent changes in immediate-early gene expression which ultimately increases gonadotropin synthesis [31]. We examined the impact of Gas6-dependent AXL receptor activation on ERK phosphorylation, expression of the transcription factor Egr-1 (early growth response factor 1) in αT3-1 and LβT2 cells, and production of the gonadotropin subunit LHβ in LβT2 cells. Using a semiquantitative ELISA approach, we found that Gas6 (100 nM for 3 h) increased the proportion of phosphorylated ERK relative to total ERK (pERK/ERK) in clonal gonadotrope cell cultures (Fig. 3A). To further characterize Gas6/AXL-dependent ERK phosphorylation, we incubated LβT2 cells with Gas6 (100 nM) for increasing amounts of time; we incubated LβT2 cells with GnRH (10 nM for 5 min) as a comparative positive control. Unlike GnRH, which produced an effect at 5 min, Gas6 did not increase pERK/ERK for incubation periods < 2 h (Fig. 3B). The increase in pERK/ERK elicited by Gas6 at 2 h was not different from that produced by GnRH at 5 min. Of note, although Gas6 at 5 min had no observable effect, simultaneous incubation of LβT2 cells with Gas6 and GnRH for 5 min increased pERK/ERK to a greater extent than GnRH alone (Fig. 3B).

To determine the impact of AXL receptor activation on the expression of Egr-1, we incubated αT3-1 and LβT2 cells with Gas6 (100 nM) for durations ranging from 5 min to 1 h and measured Egr-1 transcript levels with droplet digital PCR (ddPCR). For αT3-1 cells, Gas6 increased Egr-1 transcript abundance becoming evident at 20 min and peaking at 40 min (Fig. 3C). In LβT2 cells, the time course of Gas6-dependent changes in Egr-1 transcripts was faster than αT3-1 cells with an observable increase in Egr-1 transcripts at 5 min and followed quickly with a peak at 10 min.

LβT2 (but not αT3-1) cells express the gonadotropin subunit LHβ [30]. We assessed the effect AXL receptor signaling on LHβ expression by measuring protein levels in LβT2 culture media and cell lysates with ELISA. Similar to GnRH (10 nM for 2 h), Gas6 (100 nM for 2 h) increased the abundance of LHβ protein in the culture media and LβT2 cell lysates (Fig. 3D). Gas6 produced a greater increase in LHβ protein than GnRH in the culture media but not in LβT2 cell lysates. To test for potential constitutive GnRH and AXL receptors and MAP kinase activities on LHβ expression, we examined the effects of the GnRH receptor antagonist (cetrorelix; 2 nM), the AXL receptor antagonist (R428; 50 µM), and the MEK (MAPK kinase) inhibitor (U0126; 10 µM) on LHβ protein abundance (Fig. 3E). Inclusion of all three inhibitors reduced basal levels of LHβ protein in the media of LβT2 culture media to ≈ 50% of control.

### AXL enhances gonadotrope matrix metalloproteinase 9 activity and cell migration

**Fig. 4:**
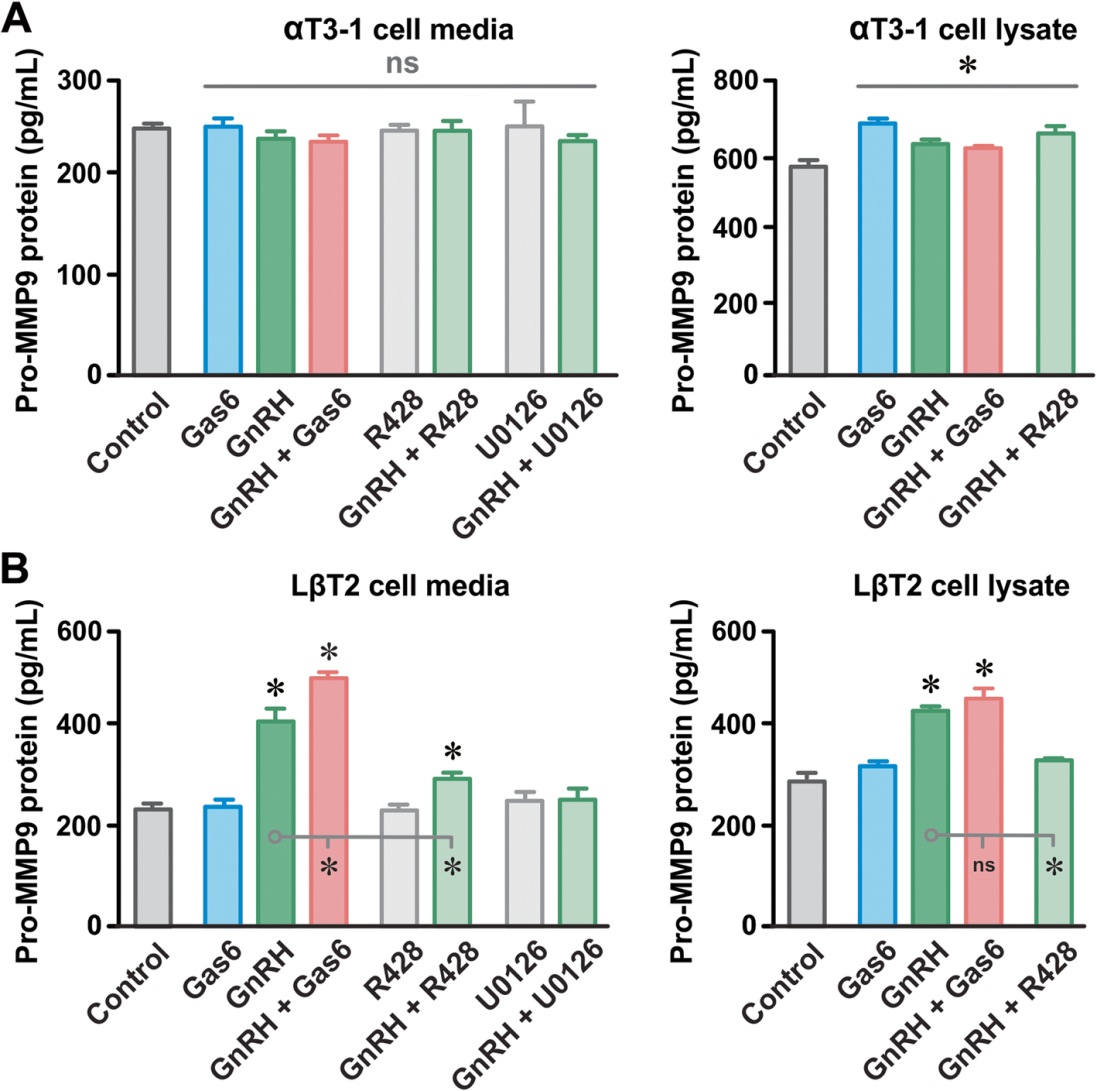
AXL and GnRH receptor stimulation promotes pro-MMP9 release. Pro-MMP9 abundance in the culture media and lysates of αT3-1 (**A**) and LβT2 (**B**) cells following incubation with Gas6 (100 nM), GnRH (10 nM), Gas6 plus GnRH, and the AXL receptor antagonist R428 (50 µM), the MEK inhibitor U0126 (10 µM). *n* = 4 for each condition. *n* = number of independent experiments; numerical data presented as mean ± SEM; ns = *P* > 0.05; * *P* < 0.05

GnRH stimulation of ERK involves the convergence of multiple signaling processes, including MMP9 (metalloproteinase 9)-dependent transactivation of EGF receptors [26]. As MMP9 and EGF receptors are known downstream mediators of AXL receptor signalling [32], and our data showing AXL-mediated potentiation of ERK above (Fig. 3A and B), we examined the effects of Gas6 on MMP9 activity in αT3-1 and LβT2 cells. To do so, we measured the abundance of the inactive pro-form of MMP9 (pro-MMP9) in lysates and culture media of αT3-1 and LβT2 cells (Fig. 4). Incubating αT3-1 cells for 24 h with Gas6, GnRH, and the MEK/ERK activator PAF C-16 produced no observable changes in pro-MMP9 levels in the culture media and a slight increase in cell lysates (Fig. 4A). For LβT2 cells, GnRH increased pro-MMP9 abundance in the culture media and in cell lysates; MEK inhibition with U0126 blocked these effects (Fig. 4B). While Gas6 produced no effect, incubating LβT2 cells with GnRH and Gas6 increased pro-MMP9 levels in the culture media than GnRH alone. Coincubation with the AXL receptor antagonist R428 attenuated GnRH-dependent increases in pro-MMP9 in LβT2 culture media and cell lysates.

**Fig. 5:**
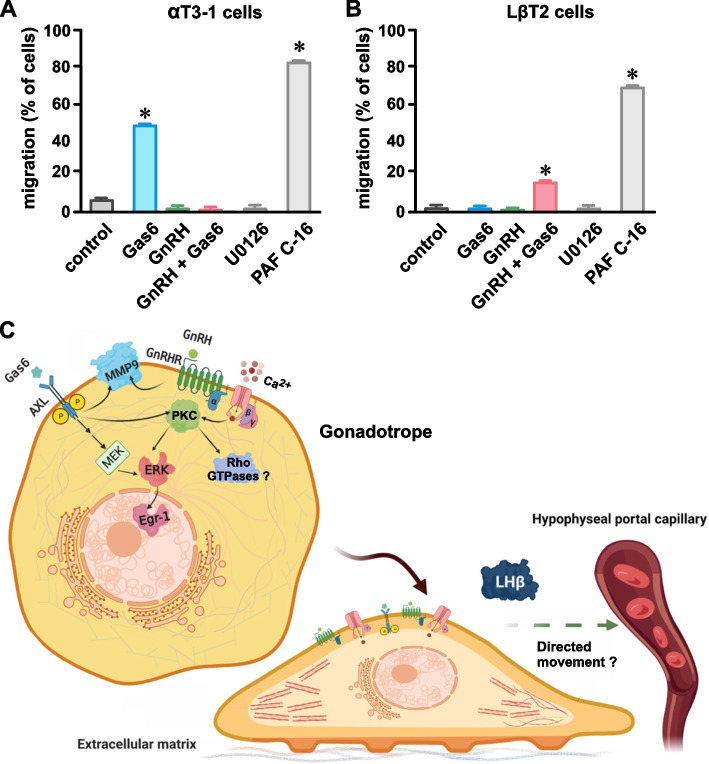
AXL and GnRH receptor activation promotes migration of clonal gonadotropes. Transwell migratory responses (% cells) of αT3-1 (**A**) and LβT2 (**B**) cells following 48 h incubations with Gas6 (100 nM), GnRH (10 nM), Gas6 plus GnRH, the MEK inhibitor U0126 (10 µM), and the MEK activator PAF C-16 (100 µM). *n* = 4 for each condition. *n* = number of independent experiments; numerical data presented as mean ± SEM; ns = *P* > 0.05; * *P* < 0.05. **C** Working model of AXL and GnRH receptor signaling integration in gonadotropes

Extracellular matrix modification by secretory endopeptidases such as MMP9 influence the migratory behavior of cells [33]. Evidence suggests that cell movement is a component of gonadotrope response to GnRH [34, 35]. Accordingly, we used a transwell cell migration assay to assess AXL-dependent modulation of αT3-1 and LβT2 cell chemotactic responses to GnRH [36]. We found that exposing αT3-1 and LβT2 cells to GnRH (10 nM) for 48 h produced no observable effects on cell movement (Fig. 5A and B). In the absence of GnRH, incubation of αT3-1 cells with Gas6 provoked an increase in cell migration. In contrast, Gas6 did not promote LβT2 cell migration except in the presence of GnRH. MEK/ERK activation with PAF C-16 (100 µM as a positive control) produced a robust increase in migration of αT3-1 and LβT2 cells while MEK inhibition with U0126 (10 µM as a negative control) had no observable effect.

## Discussion

In this paper we tested the hypothesis that AXL, one of the three TAM receptor tyrosine kinases, modulates GnRH receptor signaling. Supportive findings include: 1) demonstrable AXL expression in GnRH expressing cells (i.e., gonadotropes); 2) the AXL agonist Gas6 enhanced GnRH receptor-dependent ERK phosphorylation; 3) Gas6 increased transcriptional expression of the GnRH-responsive immediate early gene Egr-1; and 4) Gas6 enhanced production of the gonadotropin subunit LHβ. Additional observations suggesting potential of GnRH receptor function by AXL include increased pro-MMP9 synthesis and secretion and facilitated migration of clonal gonadotropes following Gas6 exposure. Altogether, our data are consistent with the concept of AXL as a novel modulator of GnRH receptor signaling.

The mechanisms by which AXL modulates GnRH receptor signaling in this study are unclear. Previous reports demonstrated crosstalk between GnRH receptors and receptor tyrosine kinases, notably GnRH-dependent EGFR transactivation in immortalized murine gonadotropes and GnRH neurons [26, 37, 38]. AXL receptors have also been associated with transactivation of receptor tyrosine kinases, including EGFR [17]. EGFR transactivation by GnRH required release of MMP2 and MMP9 [26] and AXL receptor activation is associated with release of MMP2 and MMP9 [32, 39]. Our observations regarding pro-MMP9 generation by LβT2 cells suggests a potential contributory mechanism to integration of AXL and GnRH receptor signaling. Although Gas6 alone did not alter pro-MMP9 production by LβT2 cells, Gas6 enhanced GnRH-dependent release of pro-MMP9 by LβT2 cells. Further, inhibition of AXL receptors with R428 attenuated the stimulatory effect of GnRH on pro-MMP9 generation. Together, these results provide a potential mechanism contributing to functional interactions between AXL and GnRH receptor signaling processes.

Our pERK/ERK measurements revealed that Gas6 increased ERK signaling independent of GnRH only after Gas6 incubation of 2 h. Although acute short-term (5 min) Gas6 exposure produced no observable effect on pERK levels, in the presence of GnRH, the same 5 min Gas6 exposure enhanced pERK beyond that observed with GnRH alone. The molecular basis for this facilitation of GnRH ERK signaling by Gas6 is not apparent and will require additional experimentation. Somewhat analogously, our time course experiments with Gas6 showed observable pERK/ERK changes in LβT2 cells only after 2 h of incubation. However, using ddPCR to measure Egr-1 transcripts, we found that only 5 min of Gas6 exposure was required to produce significant elevations in transcript levels. Abundant prior evidence indicates that in gonadotropes, including LβT2 cells, Egr-1 transcripts are regulated by ERK activation [31, 40–43]. Accordingly, we suggest that the underlying cause of this second apparent disconnect likely arises from large differences in the sensitivities of our pERK/ERK measurements with ELISA and Egr-1 measurements with ddPCR.

GnRH receptor signaling in gonadotropes classically originates with Gα_q_ protein-dependent activation of PLCβ [44]. The clonal murine gonadotropes used in this study recapitulate many, but not all, of the aspects of GnRH receptor signaling ascribed to native gonadotropes [29, 30]. Thus, our observations following AXL receptor manipulation in αT3-1 and LβT2 gonadotropes need to be investigated using a more biologically relevant approach. In human cancers, some of which overexpress AXL [9], GnRH receptor signaling is predominantly mediated by Gα_i_ proteins [45]. In these cells, GnRH reeptor activation produces antiproliferative effects by increasing protein tyrosine phosphatase activity leading to subsequent dephosphorylation and inactivation of EGF receptors. As such, the effects of enhancing or inhibiting AXL-dependent modulation of GnRH receptor signaling mechanisms are likely to differ from gonadotropes and should be the focus of further study.

Suggesting potential functional importance, we found that incubating LβT2 gonadotropes with Gas6 for 2 h potentiated GnRH-dependent release of LHβ into the culture medium. However, Gas6 alone produced no observable effects on the amount of pro-MMP9. Of note, we also found that AXL inhibition with R428, in the absence of Gas6, reduced basal LHβ release. The decrease in LHβ release was robust (≈ 50% control) and was comparable to reductions in basal release produced by the GnRH receptor antagonist cetrorelix and the MEK inhibitor U0126. AXL receptors undergo Gas6-independent activation via homotypic binding of extracellular domains of AXL receptors located on the surface of adjacent cells [46]. We postulate that basal AXL signaling, maintained by AXL receptor interactions between adjacent LβT2 cells, could underlie the reduction in basal LHβ release following AXL inhibition. Related to and expanding on this supposition, we found that Gas6 produced a chemotactic migration response in LβT2 cells in the presence of GnRH. Thus, Gas6 could promote AXL activation directly and indirectly by increasing the occurrence of cell–cell interactions. In this scenario, coincident exposure of LβT2 cells to Gas6 and GnRH would be expected to increase downstream signaling cascades to a greater extent than either compound alone. Our data showing enhanced GnRH-dependent ERK signaling and pro-MMP9 generation by Gas6 under conditions where the later showed no apparent effect on its own is consistent with this hypothesis.

Although the contribution of AXL to GnRH neuronal migration from the olfactory placode to the forebrain during development is established, a role for AXL in the pituitary is not evident in prior publications [5, 6, 47]. These studies, which used knockout models, conclude that AXL function in the hypothalamus, but not the pituitary, is required for reproductive function. However, our imaging experiments show clear AXL-like immunoreactivity in GnRH receptor positive cells in human and murine pituitary sections. Indeed, nearly all of the cells visualized with GnRH receptor-like immunoreactivity were also identified as AXL positive. In these pituitary sections we also visualized Gas6-like immunoreactivity, not in GnRH receptor positive cells, but in adjacent cells of unknown identity. Interestingly, published RNA sequencing data from male and female murine gonadotropes show AXL expression in these cells [48]. These data revealed apparent differential expression of AXL in female gonadotropes with greater AXL levels in cells harvested during proestrus, when GnRH pulse frequency is high, than in cells harvested during diestrus, when GnRH pulse frequency is low. Consistent with these data, we found that incubating clonal gonadotropes with GnRH produced a slight increase in AXL expression. Future investigations thoroughly examining functional relationships between GnRH receptor and AXL signaling in gonadotropes are necessary to address this issue.

## Conclusions

In sum, our data consistently supports the concept of AXL receptors as a novel modulators of GnRH receptor signaling (Fig. 5C). However, our observations are not without incongruities, such as the temporal discord between changes in ERK signaling and Egr-1 transcription and disparities between in αT3-1 and LβT2 cells. We suggest that these apparent disconnects arise not only from the many differences between αT3-1 and LβT2 cells [29, 30], but also the intrinsic complexities of AXL and GnRH receptor signaling mechanisms [16]. Additionally, the less-than-optimal efficacy and tolerability of current medications manipulating GnRH receptor expressing cells (e.g., gonadotropes) supports the need for novel pharmacotherapeutic targets. Finally, as AXL receptors are at present the target of numerous novel anticancer agents undergoing clinical trials [9, 49–51], it is clinically imperative that AXL receptor biology in non-neoplastic cells be better understood.

## Data Availability

All data generated or analyzed during this study are included in this published article.

## Abbreviations

GnRHR: Gonadotropin-releasing hormone receptor
HPG axis: Hypothalamic-pituitary–gonadal axis
LH: Luteinizing hormone
FSH: Follicle stimulating hormone
MMP9: Metalloproteinase 9
Egr-1: Early growth response factor 1
Gas6: Growth arrest-specific protein 6
PI3K: Phosphatidylinositol 3-kinase
PKC: Protein kinase C
MAPK: Mitogen-activated protein kinases
EGFR: Epidermal growth factor receptor
PDGFR: Platelet derived growth factor receptor
ERK: Extracellular signal-regulated kinase
PLC: Phospholipase C
PIP_2_: Phosphatidylinositol-4–5-bisphosphate
IP_3_: Inositol-1,4,5-trisphosphate
DAG: Diacylglycerol
ER: Endoplasmic reticulum
JNK: C-Jun N-terminal kinase
DMEM: Dulbecco’s Modified Eagle Medium
